# Artificial intelligence–enabled social media listening to inform early patient-focused drug development: perspectives on approaches and strategies

**DOI:** 10.3389/fdgth.2024.1459201

**Published:** 2024-11-20

**Authors:** Erica Spies, Jennifer A. Flynn, Nuno Guitian Oliveira, Prathamesh Karmalkar, Harsha Gurulingappa

**Affiliations:** ^1^ Work Completed While Employees of EMD Serono Research & Development Institute, Inc., Billerica, MA, United States; ^2^ Healthcare Business of Merck KGaA, Darmstadt, Germany; ^3^ Merck IT Centre, Merck Data & AI Organization, Merck Group, Bangalore, India

**Keywords:** artificial intelligence (AI), social media listening (SML), patient focused drug development (PFDD), unmet needs, machine learning (ML), deep learning (DL)

## Abstract

This article examines the opportunities and benefits of artificial intelligence (AI)–enabled social media listening (SML) in assisting successful patient-focused drug development (PFDD). PFDD aims to incorporate the patient perspective to improve the quality, relevance, safety, and efficiency of drug development and evaluation. Gathering patient perspectives to support PFDD is aided by the participation of patient groups in communicating their treatment experiences, needs, preferences, and priorities through online platforms. SML is a method of gathering feedback directly from patients; however, distilling the quantity of data into actionable insights is challenging. AI–enabled methods, such as natural language processing (NLP), can facilitate data processing from SML studies. Herein, we describe a novel, trainable, AI-enabled, SML workflow that classifies posts made by patients or caregivers and uses NLP to provide data on their experiences. Our approach is an iterative process that balances human expert–led milestones and AI-enabled processes to support data preprocessing, patient and caregiver classification, and NLP methods to produce qualitative data. We explored the applicability of this workflow in 2 studies: 1 in patients with head and neck cancers and another in patients with esophageal cancer. Continuous refinement of AI-enabled algorithms was essential for collecting accurate and valuable results. This approach and workflow contribute to the establishment of well-defined standards of SML studies and advance the methodologic quality and rigor of researchers contributing to, conducting, and evaluating SML studies in a PFDD context.

## 1 Introduction

There is increased recognition that patients’ perspectives have not always been factored into therapy research and development, clinical trial development and execution, labeling, and access to emerging therapies. Given the importance of patients’ varied health and therapy experience, this lack of inclusion has spurred a new effort to incorporate patient perspectives throughout the drug development life cycle (1). This focused effort on patient perspectives and the incorporation of patient-experience data is facilitated by traditional approaches (e.g., patient interviews, patient engagement meetings), as well as the increased participation of patient groups in communicating their treatment experiences, needs, and preferences through online platforms. Regulatory agencies have recognized the importance of including patient-focused drug development (PFDD) in the overall drug development process and have begun providing guidance on incorporating patient and caregiver perspectives to improve the quality, relevance, safety, and efficiency of drug development, and to inform drug evaluation and regulatory decision making (2–4). PFDD approaches can give patients and caregivers a voice in the drug development process by elucidating aspects of a target product value profile, identifying patient needs and preferences, and selecting clinical study endpoints (5). Various methods of gathering patient input have been endorsed by the US Food and Drug Administration (FDA), including social media listening (SML) (3, 6).

**Figure 1:**
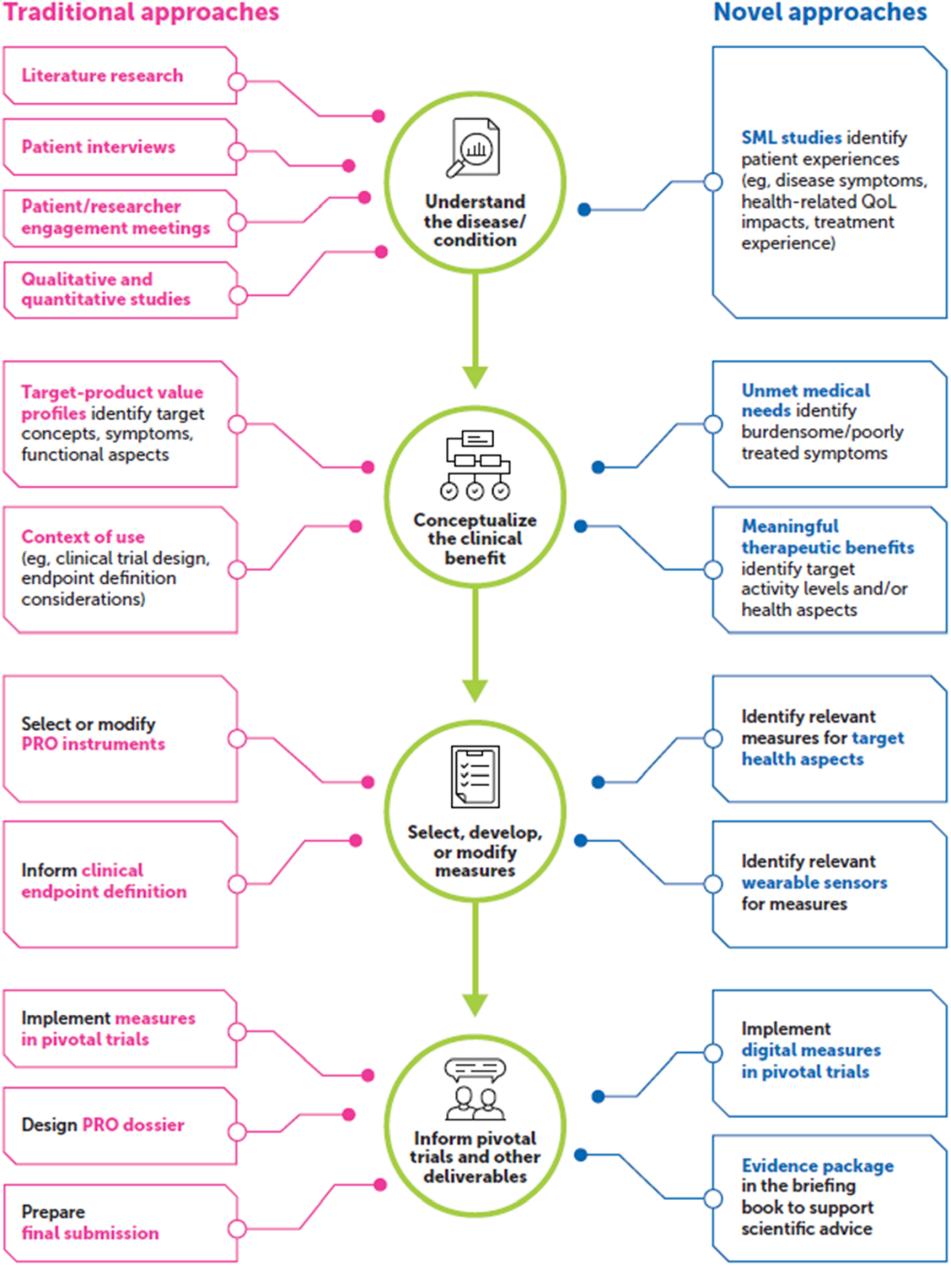
SML methods can supplement traditional PFDD approaches. PFDD, patient-focused drug development; PRO, patient-reported outcome; QoL, quality of life; SML, social media listening.

SML involves collecting and analyzing online statements and conversations from social media platforms (e.g., X®, Facebook®), patient-centric social media platforms, blogs, forums, and other means of online communication (7–9). A growing number of patients are taking to online communities and platforms to describe their individual health care journeys, acting as an invaluable source of qualitative data on unique patient perspectives that may otherwise go unheard by health care providers and drug developers. SML approaches in PFDD have been reported in numerous use cases, including identifying unmet medical needs, characterizing target patient populations, repurposing drugs, recruiting patients, detecting adverse events (AEs), and detailing patient experiences with a disease and/or treatment (10–13). SML offers the potential to gather a range of viewpoints directly from patients without compromising personally identifiable information to supplement/complement more traditional PFDD methods. See Figure 1 for an overview of how novel approaches can support traditional PFDD approaches throughout the drug development life cycle.

It is important to understand that patients’ lived experiences vary and often extend beyond treatment efficacy. Prior analyses have found that this is particularly true among patients receiving oncologic therapy, where individual experiences depend on specific cancer type/stage, therapy choice, and the presence/absence of other conditions. During a 1-h, web-based breast cancer support group, patients noted diverse concerns, including communication gaps between patients and caregivers, timeliness of insurance company responses, poor integration of palliative care, and financial challenges (14). An analysis of social media posts by patients with breast cancer found further variation in topics, including treatment patterns, patient journeys, burden of illness, unmet needs, AEs, quality of life (QoL), social impacts from the disease, and perceptions on drug effectiveness (15). Another interview-based study highlighted the importance of advanced care planning and the need for earlier discussion between health care providers and patients and families (16). These reports emphasize the importance of gathering multidimensional perspectives from diverse populations that can collectively provide valuable insights to inform treatment decisions.

In this article, we discuss existing evidence of SML application in oncology-based studies and their contributions to PFDD. We highlight the value of a novel, flexible, artificial intelligence (AI)–enabled SML workflow and describe how it can be used to gather patient and caregiver insights that can be used to promote and support PFDD. We report the approach and methodologic learnings from 2 oncology case studies (head/neck and esophageal cancers) to inform future implementations of our workflow and PFDD investigations.

## 2 Review of SML studies in oncology and their uses for PFDD

SML studies have been employed in multiple therapeutic areas, with the goal of gaining insight into the needs, motivations, behaviors, and considerations of patients and caregivers to inform early drug development strategies (7, 17–19). The oncology space is well suited for SML approaches because cancer entails a high symptom and treatment burden, with patient communities sharing experiences frequently and in detail. In 2005, more than 400,000 internet cancer support groups were found, each having approximately 2,000 members (20). In 2011, 620 breast cancer groups (fundraising, awareness, product/service, patient/caregiver support) were identified on Facebook®, with more than 1,000,000 members collectively (21). In 2020, 123 apps oriented to patients with cancer were identified across 2 major application marketplaces (22). With increasing smartphone access and a continually growing prevalence and utilization of online platforms, online platforms that address the patient and caregiver experience have become a valuable resource in SML.

Several publications have established the value of SML when seeking to identify patients’ unmet medical needs, better characterize the patient population, detect AEs, and examine treatment-switching behaviors. Arun et al. investigated the patient experience among patients with follicular lymphoma using SML and found that the most common topics discussed were the disease and treatment impacts on QoL, curability, and the fear of relapse/progression (18). Chauhan et al. employed SML to elucidate perceptions and needs among patients with melanoma in multiple European countries, further validating SML as a tool to gather evidence from patients and stakeholders across countries. Twitter® (now X®) was the primary channel across the majority of the countries examined, and the most frequently discussed topics were treatment stage, diagnosis and tests, surgery and immunotherapy, QoL issues (particularly the emotional burden of disease), and the availability of effective treatments and access to good health care providers (19). Mendelson et al. collected data from Twitter® (now X®), Facebook®, forums, and news/blogs among patients with diffuse large B-cell lymphoma, revealing that the most discussed symptoms were pain, enlarged lymph nodes, B symptoms, and fatigue. Most posts mentioned switching treatment to stem cell therapy following multiple experiences of relapse and chemotherapy failures (7).

The quantity of available social media data creates challenges to data extraction and analysis. Typically, online aggregators are employed to collect bulk social media data that are refined via natural language processing (NLP) algorithms (10, 11). Common NLP strategies include frequency and co-occurrence analysis, part-of-speech tagging, sentiment analysis, indexing/extracting key terms, term frequency-inverse document frequency, global vectorization word embedding, sequential data processing deep learning, and bidirectional encoder representation (11, 23–26). AI, or machine learning (ML), methods have also been used to process the vast quantities of data produced through social media mining in disease-specific and nonspecific social media communities for a variety of therapeutic areas beyond oncology, including dry eye disease and atopic dermatitis, where approaches such as the skip-gram negative sampling variant of the word2vec neural network and the Biterm Topic Model have been employed (12, 27, 28).

Several studies have described AI with NLP approaches for probing patient-experience data among patients with cancer. Fang et al. explored the application of NLP methods to classify unstructured text from patient interviews and identify patient-reported symptoms and QoL impacts among patients with hepatocellular carcinoma, biliary tract cancer, and gastric cancer. The most promising NLP model identified was a Bidirectional Encoder Representations from Transformers (BERT) model, which outperformed other models in accurately predicting the multiclass classification of unstructured text, demonstrated high predictive performance and could better capture the context of a word given its relative position within a sentence (26). This predictive performance was consistent across interviews from different patient populations and studies, indicating both versatility and generalizability. In summation, this proof-of-concept study showed the potential of NLP-based models to accurately contribute to the automated processing of patient interview transcripts, which could facilitate the incorporation of patient input into the drug development process. Although there are numerous examples demonstrating the value, approaches, and uses of SML, few describe a customizable workflow to conduct SML studies based on an iterative process driven by human experts and enabled by AI methods to facilitate concept identification.

## 3 Overview of an AI-enabled SML workflow

We have developed an AI-enabled SML model workflow for identifying and processing publicly available social media posts to better understand patient and caregiver experiences. This customizable workflow integrates human milestones and AI-enabled methodologies, with human experts retraining models to iteratively validate and optimize the AI-enabled methods identifying posts from patients and caregivers.

At project initiation, human experts develop key research questions, identify inclusion/exclusion criteria, and establish data sources. This is followed by gathering and processing SML data from online platforms, supported by AI-enabled screening and classification methods that use a retrainable ML model. Outputs from AI-enabled processes are reviewed throughout the workflow by human experts who provide feedback to iteratively refine the proprietary AI-enabled methodologies to further improve the signal-to-noise ratio. A sufficiently trained ML model can then be employed for each concept of interest based on predefined research question(s), providing relevant posts for analysis via NLP identification of qualitative data for synthesis of data visualization. Further, the insights and analyses produced from these steps can be leveraged to further refine the workflow and address key research questions. The Supplementary Material provides a detailed description of the consecutive steps and a general overview of this flexible, iterative, and configurable workflow that involves human-led initiatives, AI-enabled steps, and steps of iterative refinement. Given the broad array of online platforms dedicated to oncology and patients with cancer, as described earlier, we propose using this process to identify records from patients with cancer and caregivers of interest.

## 4 Oncology case studies using the AI-enabled SML workflow

To explore the applicability of this workflow, we conducted 2 studies in oncology: 1 in patients with head and neck cancers and another in patients with esophageal cancer. The complete methods and key findings from these case studies are reported elsewhere (29). In brief, we explored the following research questions: (1) What are patients’ expectations relating to treatments, and which treatment attributes are most important to patients? and (2) Which symptoms do patients find most burdensome? We then established social media sources of interest (e.g., forums, blog posts, WordPress posts (30), X® (31)) from 7 countries (United States, United Kingdom, Japan, Germany, Spain, France, and Italy), including all languages, with non-English posts translated into English. Identified posts were analyzed by the AI-enabled algorithm and classified as patient, caregiver, or irrelevant. Human experts then reviewed the posts and filtered irrelevant posts with specific tags to refine the AI-enabled algorithm. Relevant posts were then exported to ATLAS.ti for qualitative coding (32).

Through these pilot studies, we found that our customizable AI-enabled workflow, with iterative human expert–led training and ML, effectively identified patient and caregiver posts to reveal valuable concepts related to our research questions. Specifically, the workflow identified relevant posts relating to the most frequent products/treatment categories, potential AEs, symptom severity, and psychosocial health. We note that iterative engagement of experts with the AI-enabled methods was essential to refine algorithms and enhance the utility of the workflow.

## 5 Limitations and challenges encountered while using the AI-enabled SML workflow

Several limitations impacted the insights gained with this SML approach. Although it was possible to gather relevant data by indication and treatment type, the specific type and/or name of chemotherapy treatment was rarely detailed in posts. Additionally, when posts included >1 treatment type, it was difficult to determine which descriptions were associated with an individual treatment type, confounding how distinct therapies correlate with specific patient experiences. Further, patients’ expectations were based on various points of reference, such as information provided by their care team, online sources or other social media posts, and/or fear of the side effects of cancer treatment Therefore, interpretation of the patient experience would be impacted and possibly biased by individuals’ prior experiences.

Throughout the qualitative analysis processes, we discovered that it was challenging to distinguish between cancer-related symptoms and treatment-related symptomatic AEs. Patients frequently posted about symptomatic AEs and the burden from treatment; however, at times, it was unclear whether those symptoms were also cancer related and, if so, it was challenging to associate specific symptomatic AEs with specific treatments. Despite the utility of our novel and customizable workflow, there remained a high proportion of irrelevant entries, even in posts tagged with high confidence by the AI-enabled algorithm, and it was difficult to determine how many patients experienced symptoms out of a total population given repeat or follow-on posts. Supplementary Table 1 includes sample posts, with ATLAS.ti coding visualizations, and an overview of challenges faced when interpreting the posts.

Previous reports of SML studies have identified additional limitations with SML strategies, including the self-selection bias among those who post on social media, as patients who choose to do so may not be representative of the entire target group (5, 9–11, 33). Thus, some patient groups may be underrepresented in social media–based analyses, such as older patients, low literacy patients, and patients from some geographic regions (10, 13). Additionally, negative perceptions may be posted more commonly than positive experiences, potentially skewing the evaluation of a given issue (11). Determining the volume of data needed is another challenge with SML. For instance, fewer posts may be needed to gather data on breast cancer than on rare diseases, given high occurrence rates and public awareness. A scoping review of 77 documented uses of AI-based SML by Tricco et al. reported a median (range) duration of data collection of 1.13 years (6 months–7 years) and a median (range) number of social media posts of 42,594 (4608-711,562) (34). These ranges highlight the high variability of data volume used in SML efforts.

## 6 Discussion

This article describes the value and utility of our newly developed, AI-enabled SML workflow. We describe learnings using 2 oncology case studies that produced descriptions of patient experiences with varying degrees of granularity, including high-level list-type descriptions of treatment regimens, detailed treatment experiences, symptomatic AEs, the impact of treatment on day-to-day life, and psychosocial impacts.

Incorporating evidence and insights from SML can elevate patients’ voices in PFDD, adding valuable perspectives that may go unheard when using more traditional methods, such as patient and caregiver advisory boards, evidence-generation plans, and patient-centered outcome endpoints. Although prospective, qualitative patient interviews are the gold standard for understanding health impacts directly from patients, SML studies can provide greater geographic range and more varied input, and can be used synergistically with patient interviews to enhance the confidence of the gathered input (35, 36). Further, as SML methodologies continue to improve, a greater range of patient input can be incorporated into PFDD.

This SML workflow may be particularly useful when investigating therapies with high impacts on QoL or daily functioning, as is the case for many cancer treatments that lead to most patients experiencing side-effect profiles that would be unacceptable for other non–life-threatening diseases. New drugs continue to be developed across cancer indications, and our AI-enabled SML workflow has the potential to generate key patient/caregiver data. This information may be directly implemented to address the unique challenges of cancer treatments (e.g., identifying emerging unmet needs, how disease/treatment impacts daily living, the preferred method of drug administration, time requirements/how long patients are willing to spend at a doctor's office, and what patients may prioritize when choosing treatments).

Ideally, in drug development, researchers collect this type of data on patients’ perspectives and integrate this information with data on treatment outcomes, ultimately providing all data to the FDA during regulatory review. When successfully executed, this process results in the approval of new medicines that are both efficacious and reflective of information that is meaningful to patients, caregivers, and providers (37). Furthermore, this workflow could be used to collect real-world data and identify perspectives from minority groups that are often excluded from clinical trials, which can be submitted as evidence to the FDA. Finally, the workflow may have added utility in collecting important postmarked surveillance data, such as patient satisfaction, patient compliance, and concerns that may interfere with future success (patient trust, insurance coverage).

The workflow reported here for AI-enabled SML offers a model for iterative engagement through the initial quality check/classification, continuous review of the AI-enabled outputs, and examination of the qualitative analysis. Furthermore, human-led fine-tuning will allow this workflow to adapt with the evolving needs and expectations of patients, thus priming AI-based SML models to extract data efficiently and effectively in an automated manner without losing that human guidance. Continued use of the dashboard in real-world settings further improved quality, and this approach has potential value in multiple patient populations of interest.

**Figure 2:**
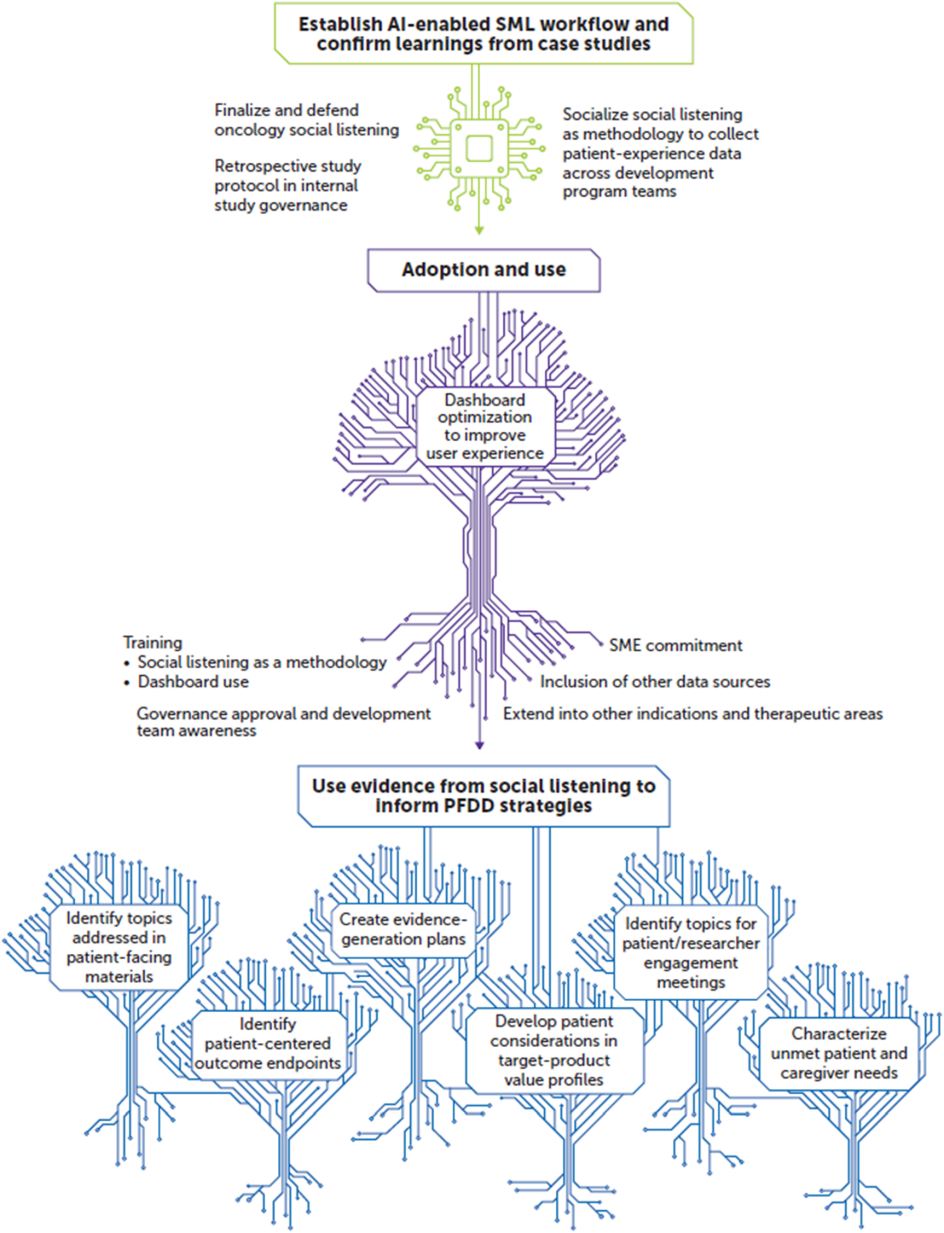
Future outlook: working toward a patient-experience knowledge hub. AI, artificial intelligence; PFDD, patient-focused drug development; SME, subject matter expert; SML, social media listening.

There are multiple future directions for this AI-enabled SML workflow. One is developing qualitative analysis plans that inform the drug development process in a way that prioritizes meaningful outcomes for patients and caregivers. We are also exploring the capture of quantitative metrics that incorporate patient data or involve the patients themselves in dashboard development and algorithm refinement. The implementation of patient-centric terms, concerns, and conversational tone may allow for a better understanding and interpretation of context within patient or caregiver posts. Figure 2 shows a conceptual overview on the benefits and uses derived from our AI-enabled SML workflow, its adoption and use, and its ultimate implementation to inform PFDD strategies.

Utilizing AI-enabled models to gather patient-focused data from the plethora of social media platforms and to categorize large amounts of data is an opportunistic strategy; however, methods require frequent fine-tuning due to the constant evolution of patient needs and expectations and technological advances. Our approach advances the tools available for SML studies and adds to the methodologic quality and rigor from the perspective of researchers contributing to, conducting, and evaluating SML studies in a PFDD context. This perspective manuscript provides further evidence on the utility of SML approaches to gain information on patients’ experiences, which align with guidance offered by the FDA and the European Medicines Agency authorities for PFDD. Using SML to inform PFDD complements traditional methods of gathering patient input and enhances many aspects of drug development.

## Data Availability

The raw data supporting the conclusions of this article will be made available by the authors, without undue reservation.
