# Supplementary material for: Artificial intelligence–enabled social media listening to inform early patient-focused drug development: perspectives on approaches and strategies
Source: Front Digit Health. 2024 Nov 20;6:1459201. doi: 10.3389/fdgth.2024.1459201 (PMC11614768; doi:10.3389/fdgth.2024.1459201)
Supplement: Supplementary file 1 [file Datasheet1.docx]

Supplementary Material

# Overview and Steps of the artificial intelligence (AI)–enabled Social Media Listening (SML) Workflow.

Consecutive steps of this AI-enabled SML workflow are shown in **Supplemental Figure 1** and include the following steps:

1. Define the research question
   1. The workflow is initiated by defining research questions, which may explore how patients experience disease symptoms/severity, its impacts (physical, psychosocial, functional, and/or health-related quality-of-life effects), or therapies, and by identifying relevant data sources (ie, social media sites, geographic regions, and languages)
2. Data wrangling and preparation
3. Data preprocessing
4. Patient and caregiver classification
   1. AI-enabled methods can support data preprocessing (ie, relevancy screening) and patient and caregiver classification using a retrainable machine learning (ML) model. Human experts then review the AI-enabled outputs to verify the accuracy of relevancy screening and patient/caregiver tagging and provide feedback for refining the AI-enabled methodologies to further improve the signal-to-noise ratio
   2. Patient-reported: A record is considered to be relevant when there is evidence that the author of the post is living with or has suffered from the disease of interest
   3. Caregiver-reported: A record is considered to be relevant when there is evidence that the author of the post is or has been looking after a patient living with or suffering from the disease of interest, regardless of the patient-caregiver familial relationship. The only recommended exceptions are records reported by patients’ spouses due to their expected daily proximity with the patient (ie, only spouses were considered relevant caregivers even if there was no explicit evidence of caregiving)
5. AI with NLP workflow
   1. Screen a sample of social media data (ie, records or posts from social media datasets of interest) and manually categorize records as relevant or irrelevant based on the previous criteria. Supply the machine with further details, when relevant, to facilitate and accelerate the learning process
   2. Apply the ML algorithms to a large social media dataset and assess the results. Repeat step 2 until the machine can categorize records with a confidence score in the range of 0 to 1. The confidence score is calculated to determine the likelihood of the predicted label matching the user’s expectation; it is the probability of the label being correctly assigned by the ML models (1)
   3. Train the ML process for each concept of interest based on predefined research question(s) by adapting steps 1 to 3 and create a dashboard for each to allow tailored analyses. Examples of concepts of interest to consider include the following:
      1. Symptoms associated with a specific disease: Which symptoms do patients find most burdensome?
      2. Experience with treatment(s): What is the level of satisfaction with the current treatment options/standard of care?
      3. Unmet needs: From the patient perspective, what do current treatments fail to address?
6. Data visualization
   1. Relevant posts are analyzed via natural language processing methods to identify qualitative data, resulting in the synthesis of data visualization
7. Data analyses and reporting
   1. Insights and analyses produced from the steps above can be leveraged to further refine the workflow or address the key research questions
   2. ML algorithms can learn patterns in the data identified by health outcomes research experts and apply those patterns to large datasets
   3. It is suggested that clear operational definitions of patients, caregivers, and concepts of interest be established as a way to create consistent methodologic standards across SML studies

# Supplementary Figures and Tables

**Supplementary Figure 1.** Overview of an AI-enabled SML solution to inform PFDD strategies. Purple indicates human-led initiatives, green indicates AI-enabled steps, and pink arrows indicate steps of iterative refinement in the workflow. AI, artificial intelligence; ML, machine learning; NLP, natural language processing; PFDD, patient-focused drug development; SML, social media listening.


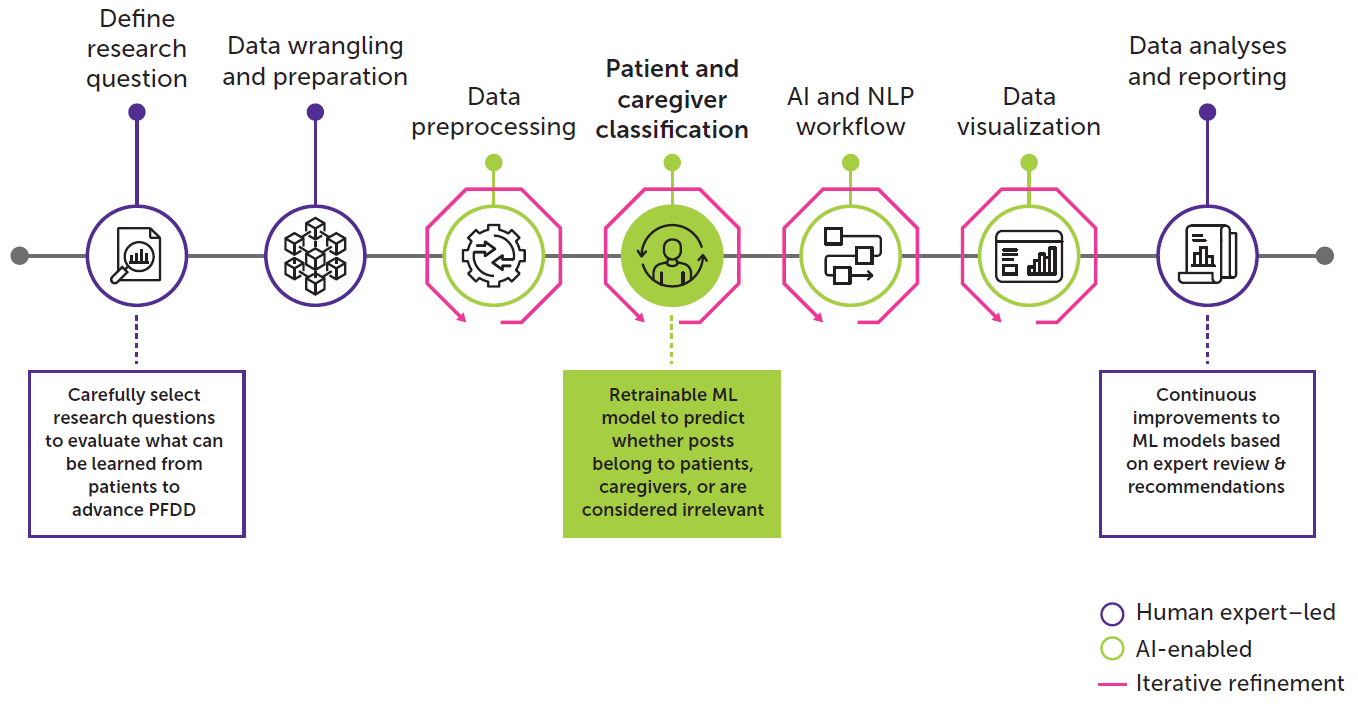


**Supplementary Table 1.** Example posts and ATLAS.ti coding visualization of qualitative analysis from patients with head, neck, or esophageal cancers. AE, adverse event; AI, artificial intelligence; chemo, chemotherapy; RT, radiation therapy; SML, social media listening; Tx, treatment.

| **#1: Example post identified by AI-enabled SML workflow** | **ATLAS.ti coding visualization** |
| --- | --- |
| “I was diagnosed with Tonsil cancer a couple of months ago and Friday was day 13 of radiotherapy so on Tuesday I will already be 1/2 way I had Chemo on day 1 and will have 1 more on 25th. I felt a bit 'yuk' after the chemo but not too bad and was doing ok until day 10 when my tongue was sore. The last few days got quite bad quite quickly. I'm struggling to eat but still managing if I persevere (my wife is making sure I do!). Worst right now is the lack of or very thick saliva and a sore throat. Given the pain now, I'm trying not to think how much worse it will get. I've tried to read as much of this thread as possible and appreciate all the info and advice.” | AE: chemo: sore tongue/throat  AE: chemo/RT: difficulty eating  AE: chemo/RT: lack of or thick saliva  Tx expectations: chemo/RT: worsens with time |
|  | **Challenge** |
|  | - Can be difficult to distinguish whether patients are talking about cancer symptoms or treatment side effects - Type/name of chemotherapy is rarely provided in detail |
| **#2: Example post identified by AI-enabled SML workflow** | **ATLAS.ti coding visualization** |
| “I usually have early dumping syndrome symptoms but occasional have late (or delayed) dumping syndrome. I can eat whatever I want, but I can only eat a very limited mount of food at any one sitting and dumping syndrome occurs with even a small amount of food …. My symptoms after eating are typically as follows: Immediate fatigue (with plenty of yawning). As mentioned above, the fatigue makes me fall asleep at times. Fullness with sight nausea sometimes. Feeling flushed and heavy breathing. Diarrhea if I eat too much.”  “It has now been 13 weeks since my final radiation (33 sessions) for my Squamous Cell Carcinoma throat cancer on the right side base of my tongue... My issue is the severe pain in my throat. I still have several mouth ulcers that are not healing. The pain in my throat is constant but exasperated by any attempt to eat or drink…Pain level varies daily (hourly). Either get burning pain while consuming liquids or sometimes it feels like swallowing razor blades.” | AE: surgery (colon interposition): diarrhea  Symptoms: eating  AE: surgery (colon interposition): nausea  Symptoms: pain  AE: radiation: pain |
|  | **Challenge** |
|  | - Can be difficult to distinguish whether patients are talking about cancer symptoms or treatment side effects |

# Supplementary Reference

1. Tonye G. (2021). Machine learning confidence scores — all you need to know as a conversation designer. Available from: <https://medium.com/voice-tech-global/machine-learning-confidence-scores-all-you-need-to-know-as-a-conversation-designer-8babd39caae7>. Accessed May 8, 2023.
